# Bone Marrow Mesenchymal Stem Cells Ameliorate Cisplatin-Induced Renal Fibrosis *via* miR-146a-5p/Tfdp2 Axis in Renal Tubular Epithelial Cells

**DOI:** 10.3389/fimmu.2020.623693

**Published:** 2021-02-16

**Authors:** Lei Wu, Chao Rong, Qing Zhou, Xin Zhao, Xue-Mei Zhuansun, Shan Wan, Mao-Min Sun, Shou-Li Wang

**Affiliations:** ^1^ Department of Pathology, School of Biology & Basic Medical Sciences, Soochow University, Suzhou, China; ^2^ Collaborative Innovation Center of Clinical Immunology, Sihong People’s Hospital, Soochow University, Suzhou, China; ^3^ Department of General Surgery, Affiliated Hospital of Jiangsu University, Zhenjiang, China; ^4^ Laboratory Animal Research Center, Medical College of Soochow University, Suzhou, China

**Keywords:** bone marrow mesenchymal stem cells, renal fibrosis, microRNA, TGF-β1, renal tubular epithelial cells

## Abstract

Mesenchymal stem cells (MSCs) have regenerative properties in acute kidney injury (AKI). However, the potential function of MSCs in chronic kidney disease remains elusive. Renal fibrosis is the common endpoint of chronic progressive kidney diseases and causes a considerable health burden worldwide. In this study, the protective effects of bone marrow mesenchymal stem cells (BM-MSCs) were assessed in repeated administration of low-dose cisplatin-induced renal fibrosis mouse model *in vivo* as well as a TGF-β1-induced fibrotic model *in vitro*. Differentially expressed miRNAs in mouse renal tubular epithelial cells (mRTECs) regulated by BM-MSCs were screened by high-throughput sequencing. We found microRNA (miR)-146a-5p was the most significant up-regulated miRNA in mRTECs. In addition, the gene Tfdp2 was identified as one target gene of miR-146a-5p by bioinformatics analysis. The expression of Tfdp2 in the treatment of BM-MSCs on cisplatin-induced renal injury was evaluated by immunohistochemistry analysis. Our results indicate that BM-MSC attenuates cisplatin-induced renal fibrosis by regulating the miR-146a-5p/Tfdp2 axis in mRTECs.

## Introduction

Renal fibrosis is characterized by the activation of massive fibroblast and deposition of the fibrotic matrix in kidney tissue, which is considered as the common endpoint of chronic kidney disease (CKD) (1, 2). Renal fibrosis mainly affects over 70-years old adults who account for over 10% of the world population (3). In 2017, the global population of CKDs was about 6.97 million, and patients with CKD in China were approximately 1.32 million. Between 1990 and 2017, the global prevalence of CKD increased by 29.3%, and the global mortality rate increased by 41.5% (4). However, the therapeutic strategies for CKD, such as kidney transplantation or dialysis, are still limited. A shortage of donor organs impedes kidney transplantation, and the cost of dialysis becomes unsustainable, especially in developing countries (5). Therefore, exploring novel and better therapeutic approaches are urgently needed.

Mesenchymal stem cells (MSCs) are multipotent cells with robust capacities of self-renewal, regeneration, proliferation, and differentiating into distinct functional cells (6, 7). MSCs can differentiate into various types of cells, such as osteoblasts, chondrocytes, or adipocytes under appropriate conditions *in vivo* or *in vitro*. Therefore, MSCs are widely used in tissue engineering, gene therapy, and immunotherapy (8, 9). The immunomodulatory and paracrine capabilities of MSCs may present the potential for therapeutic capacities in response to local environmental cues. Recent studies have reported that bone marrow MSCs (BM-MSCs) possess regenerative properties and immense plasticity to contribute to their therapeutic effects in organ injury, including acute kidney injury (AKI) and CKD (10–13). However, the mechanisms by which BM-MSCs protect renal tissues are complex, such as preventing renal tubular epithelial cell apoptosis, promoting renal tubular epithelial cell proliferation, and modulating the immune system by various cytokines and growth factors (14). MicroRNAs (miRNAs) are endogenous non-coding RNAs with a length of about 21–24 nucleotides, which regulate protein translation by combining with complementary sequences in the 3’non-coding region (3’UTR) various mRNAs (15). Recently, it has been widely accepted that miRNAs play vital roles in the progression of kidney disease (16). Specific miRNAs are associated with the initiation and development of renal fibrosis by anti- or pro-fibrogenic effects (17).

In the present study, we established a mouse model with renal fibrosis by repeated administration of low-dose cisplatin. The protective effects of BM-MSCs have been examined *in vivo* as well as in TGF-β1-treated mouse renal tubular epithelial cells (mRTECs) with an indirect co-culture environment. Next, we performed a high-throughput sequencing to identify significantly regulated miRNAs by mRTECs and probe further the underlying indirect molecular mechanism of miRNAs in the therapeutic process of BM-MSCs.

## Materials and Methods

### Ethical Considerations

Experimental protocols were approved by the Animal Ethics Committee of Soochow University and were performed following the guidelines for use and care of laboratory animals of the Experimental Animal Committee of Soochow University (Approval No : ECSU-201800099).

### Mouse Model of Renal Fibrosis

Eight-week-old male C57BL/6 mice were purchased from Zhao Yan Animals Co., Ltd. (Suzhou, China). On arrival, the mice were housed under antiviral and antibody-free micro-isolator conditions and fed a standard diet. Previous studies reported that multiple administrations of cisplatin cause renal fibrosis (18). We established and developed kidney injury and fibrosis C57BL/6 mice models using a low dose treatment of cisplatin once a week for 6 weeks. The mice were randomly divided into three groups as normal control (NC) group, cisplatin group, and cisplatin + BM-MSC group (n=6 for each group): 1) NC group: mice underwent intraperitoneal injection of 0.9% NaCl solution 10μl/g, once a week; from the 4th week underwent injection of 0.9% NaCl solution 0.4 ml through the tail vein, once per week. 2) Cisplatin group: intraperitoneal injection of 3.5mg/kg cisplatin solution according to the bodyweight of the mouse, once per week; from the 4th week, 0.9% NaCl solution 0.4 ml was injected through the tail vein once per week. 3) Cisplatin + BM-MSC group: intraperitoneal injection of 3.5mg/kg cisplatin solution according to the bodyweight of the mouse, once per week; from the 4th week, injection with normal saline containing 1.5×10^6^/ml BM-MSCs, 0.4 ml per mouse, once a week. At 6th weeks post-injection, the mice were sacrificed, the kidneys and serum from all mice were collected for further investigation.

### *In Vitro* Co-Culture Experiments

mRTECs were obtained from ScienCell Research Laboratories. BM-MSCs were gifted by Prof. Dr. Yufang Shi’s Laboratory, Soochow University. mRTECs and BM-MSCs were cultured in DMEM, low glucose medium (HyClone) with 10% FBS (Gibco), 100 units/ml penicillin, and 100 μg/ml streptomycin. All cells were maintained at 37°C in a humidified atmosphere with 5% CO_2_. mRTECs were seeded in 6-well plates at 1×10^5^/well, and TGF-β1 (10 ng/ml) was added into the medium. After 48 h of stimulation, the medium of each group was changed to a complete medium without exogenous cytokine TGF-β1. A Transwell system was selected to establish the indirect co−culture environment. BM-MSCs were added to the transwell chamber at 1×10^5^/well and indirectly co-cultured with mRTECs in a 37°C incubator for 24 h. A complete medium without BM-MSCs was added to the upper chamber for the control group.

### Histological Analysis

C57BL/6 mouse kidney tissues were sectioned and fixed in formalin. Paraffin-embedded tissues were stained with Hematoxylin and eosin (H&E), Masson trichrome, and Sirius red. Sirius red staining was performed following the Sirius Red Stain Kit (Yuanye Bio-Technology, Shanghai, China). Interstitial fibrosis areas were assessed using microscopy (Olympus) by examining five randomly selected fields (×100) of the cortex. The staining figures were evaluated independently by two pathologists and quantified by histograms.

### MiRNA High-Throughput Sequencing and Quantitative Real-Time Reverse-Transcription Polymerase Chain Reaction

High-throughput sequencing of miRNA was performed by Cloud-seq Biotechnology Co., Ltd (Shanghai, China). Isolation of miRNA from cultured cells and tissues was performed by using the Total RNA Miniprep Purification Kit (GeneMark). Grind fresh mouse kidney tissues with an electric grinder, total RNA was extracted from mRTECs, and grated kidney tissues by 1ml TRIzol and miRNA were isolated according to the manufacture’s instructions. Reverse transcription for specific miRNAs was performed using 2 μg miRNA and respective primers for reverse transcription (listed in **Supplementary Table 1**) according to the Stem-loop method. Quantitative real-time PCR was carried out using the ABsolute qPCR SYBR Green ROX Mix (Thermo Fisher Scientific) and the following cycling condition: 95°C for 10 min, 40 cycles of 95°C for 15 s, and 60°C for 60 s. StepOne Plus device (Thermo Fisher Scientific) was used for detection. The snRNA U6 was used for normalization.

### Western Blotting Analysis

The protein was extracted from fresh tissues and mRTECs by using RIPA lysate. The total protein concentration was measured using a BCA protein quantitative kit and denatured by heating. The protein samples were assessed by 10% sodium dodecyl sulfate polyacrylamide gel electrophoresis (SDS-PAGE), semi-dry transferred to polyvinylidene fluoride (PVDF), and incubated with primary antibody against α-SMA (1:350, Affinity, USA) and Col1α1 (1:500, Abcam, USA) at 4°C overnight. Next, after being washed by TBST three times and incubated with a specific secondary antibody (1:3,000; Boster, China) for 2 h at room temperature. Immunoreactivity was visualized using an ECL detection system.

### Cell Transfection

Cells were transfected with 50nM miR-146a-5p mimics/inhibitors or Tfdp2 siRNA (RiboBio Co, Guangzhou, China) when the cell density reached 80–90% prior. Lipofectamine 2000 reagent (Invitrogen, No. 1070962) was used for siRNA transfection. The cells were transferred to low glucose-DMEM with 10% FBS complete medium 6 h after transfection. Cells were harvested after 24 h.

### Immunochemistry Staining

C57BL/6 mouse kidney tissues were sectioned and fixed in formalin. After the sections were dewaxed, antigen repaired, and serum sealed, Incubate membrane and Tfdp2 primary antibody (1:200, Abcam, ab235830) overnight at 4°C. PBS was used to wash the sections, and then the samples were incubated in biotinylated anti-rabbit secondary antibody (1:200, Beyotime, China).

### Statistical Analysis

All data are expressed as the mean + standard error of the mean. Multiple comparisons were performed using Student’s *t*-test and one-way ANOVA. Statistical analysis was processed by Prism 8 software (GraphPad Software, CA). Differences between mean values were considered statistically significant at P < 0.05.

## Results

### Bone Marrow Mesenchymal Stem Cells Attenuate Renal Injury and Fibrosis *In Vitro* and *In Vivo*

**Figure 1:**
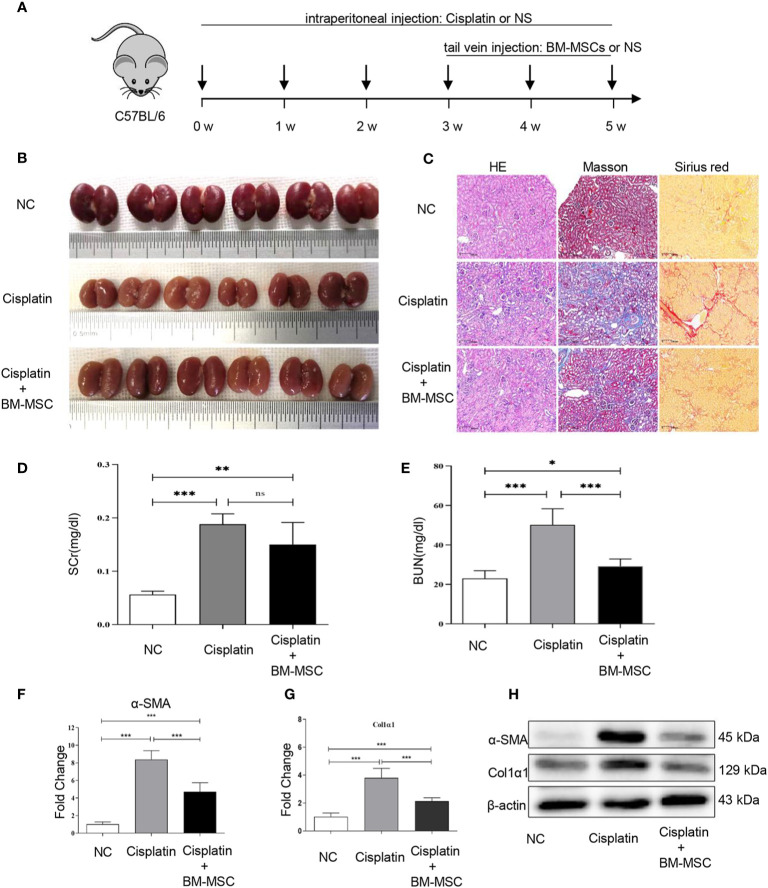
The effect of bone marrow mesenchymal stem cells (BM-MSCs) on cisplatin-induced renal injury and fibrosis *in vivo*. **(A)** The schematic diagram of mice infected with cisplatin solution intraperitoneally and suspension of BM-MSCs *via* the tail vein. **(B)** Image of the morphology of kidney in mice infected cisplatin solution intraperitoneally and suspension of BM-MSCs *via* the tail vein. **(C)** H&E, Masson, and Sirius red staining. **(D, E)** serum creatinine (SCr) and blood urea nitrogen (BUN) levels of serum in mice were determined. The relative mRNA expression levels **(F, G)** and the protein levels **(H)** of α-SMA and Col1α1 in mice kidney tissues were evaluated by real-time PCR and Western blot, respectively. Data represent the mean ± SE from three independent experiments. *P < 0.05; **P < 0.01; ***P < 0.001; NS, not significant.

We established and developed kidney injury and fibrosis C57BL/6 mice models using a low dose treatment of cisplatin once a week for 6 weeks (cisplatin group). To explore the effect of BM-MSCs on renal injury and fibrosis, mice were injected with the suspension of BM-MSCs *via* the tail vein (cisplatin +BM-MSCs group) (**Figure 1A**). All mice were euthanized at 6 weeks after the first cisplatin administration. Compared to the normal control group (NC), the volume and weight of kidneys in the cisplatin group and BM-MSCs-treated group were obviously reduced, which displayed granular or nodular surface. There was no significant difference in the appearance of the kidney between cisplatin group and cisplatin +BM-MSCs group (**Figure 1B**). To characterize this phenotype in more details, histologic and molecular tests were performed. H&E staining, Masson staining (collagen fibers marked blue), and Sirius red staining (collagen fibers marked red) showed that the fibrotic areas of the cisplatin group were significantly induced as compared to NC group kidneys. After injected the suspension of BM-MSCs *via* the tail vein, the fibrotic areas were reduced as compared with the cisplatin group (**Figure 1C**, **Supplementary Figures 1A, B**). Next, the biomarkers of kidney function and injury in serum were measured. The apparent elevation of serum creatinine (SCr) and blood urea nitrogen (BUN) levels was observed in the cisplatin group. Only the BUN level was significantly decreased by the treatment of BM-MSCs (**Figures 1D, E**). Analysis of fibrotic gene markers by RT-qPCR showed that the transcript levels of α-SMA and Col1α1 were significantly elevated in the cisplatin group compared to NC kidney tissues, while injection of BM-MSCs reduced the levels of α-SMA and Col1α1 (**Figures 1F, G**). The findings were confirmed by western blot analysis in protein level (**Figure 1H**). These results demonstrated that multiple administrations of cisplatin induced renal injury and fibrosis in mice, which could be attenuated by treatment with BM-MSCs through decreasing the expression of α-SMA and Col1α1.

**Figure 2:**
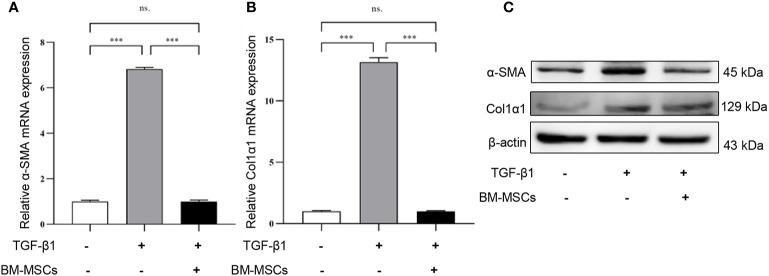
The effect of bone marrow mesenchymal stem cells (BM-MSCs) on TGF-β1-induced a fibrotic model *in vitro*. The relative mRNA expression levels **(A, B)** and the protein levels **(C)** of α-SMA and Col1α1 in mRTECs were evaluated by real-time PCR and Western blot, respectively. Data represent the mean ± SE from three independent experiments. ***P < 0.001; NS, not significant.

To verify whether BM-MSCs had a therapeutic effect on renal fibrosis *in vitro*, we used exogenous cytokine TGF-β1 to stimulate mRTECs for 48h and to establish a fibrotic model *in vitro*. Subsequently, TGF-β1-treated mRTECs were divided into two groups: with or without BM-MSCs co-cultured treatment for 24 h. The expression of α-SMA and Col1α1 in mRTECs were detected by RT-qPCR analysis in transcript level (**Figures 2A, B**) and western blot analysis in protein level (**Figure 2C**). Our results revealed that both transcriptional and translational levels of α-SMA and Col1α1 in mRTECs were significantly reduced while co-cultured with BM-MSCs, which suggested that BM-MSCs could alleviate fibrosis in mRTECs.

### Screening and Validation of Differentially Expressed miRNAs in Mouse Renal Tubular Epithelial Cells Regulated by Bone Marrow Mesenchymal Stem Cells

**Figure 3:**
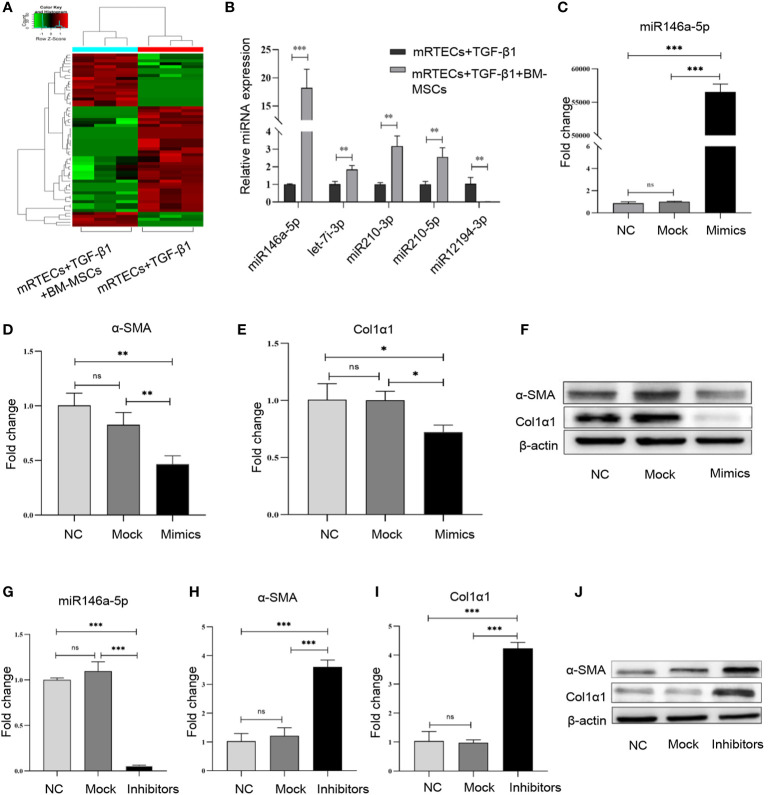
miR-146a-5p was significantly up-regulated by bone marrow mesenchymal stem cells (BM-MSCs) in mouse renal tubular epithelial cells (mRTECs). **(A)** Heatmap of differential microRNA (miRNA) expression in TGF-β1-treated mRTECs with or without BM-MSCs. Gene expression data were obtained with miRNA sequence and the most obvious values are shown. Red indicates up-regulation, while green means down-regulation. **(B)** Top-five most significant changed miRNAs were verified by real-time PCR. **(C)** TGF-β1-treated mRTECs were transfected with miR-146a-5p mimics. The levels of miR-146a-5p were determined by real-time PCR. The relative mRNA expression levels **(D, E)** and the protein levels **(F)** of α-SMA and Col1α1 in mRTECs transfected with miR-146a-5p mimics were evaluated by real-time PCR and Western blot, respectively. **(G)** TGF-β1-treated mRTECs were transfected with miR-146a-5p inhibitor. The levels of miR-146a-5p were determined by real-time PCR. The relative mRNA expression levels **(H, I)** and the protein levels **(J)** of α-SMA and Col1α1 in mRTECs transfected with miR-146a-5p inhibitor were evaluated by real-time PCR and Western blot, respectively. Data represent the mean ± SE from three independent experiments. *P < 0.05; **P < 0.01; ***P < 0.001; NS, not significant.

To screen the differentially expressed miRNAs in mRTECs regulated by BM-MSCs, we performed a miRNA high-throughput sequencing (series GSE148144) for TGF-β1-treated mRTECs with or without BM-MSCs co-culture. The heatmap of all the different expressed miRNAs was shown in **Figure 3A**. We discovered a panel of miRNAs (miR-146a-5p, miR-12194-3p, miR-210-3p, miR-210-5p, let-7i-3p) with the top-five most significant changes in two groups, which were verified by RT-qPCR analysis and revealed that miR-146a-5p was the most significant up-regulated miRNA (**Figure 3B**). According to these results, we confirmed that BM-MSCs exert the anti-fibrosis function by regulating miR-146a-5p expression in mRTECs.

To investigate the function of miR-146a-5p regulated by BM-MSCs in the progression of renal fibrosis *in vitro*, we transiently transfected miR-146a-5p mimics into TGF-β1-treated mRTECs to significantly up-regulate the expression of miR-146a-5p (**Figure 3C**). In line with the previous results, both transcriptional and translational levels of α-SMA and Col1α1 in mRTECs were significantly reduced by the miR-146a-5p mimics treatment (**Figures 3D–F**). Next, we transiently transfected miR-146a-5p inhibitors into TGF-β1-treated mRTECs to down-regulate miR-146a-5p expression (**Figure 3G**). The RT-qPCR analysis and western blot analysis showed that loss of miR-146a-5p significantly increased the expression of α-SMA and Col1α1 in mRTECs (**Figures 3H–J**). In summary, gain- or loss-function of miR-146a-5p studies by mimic and inhibitor revealed that BM-MSCs might exert its anti-fibrosis effect by increasing the expression of miR-146a-5p in mRTECs.

### Prediction and Verification of miR-146a-5p Target Genes in Mouse Renal Tubular Epithelial Cells

**Figure 4:**
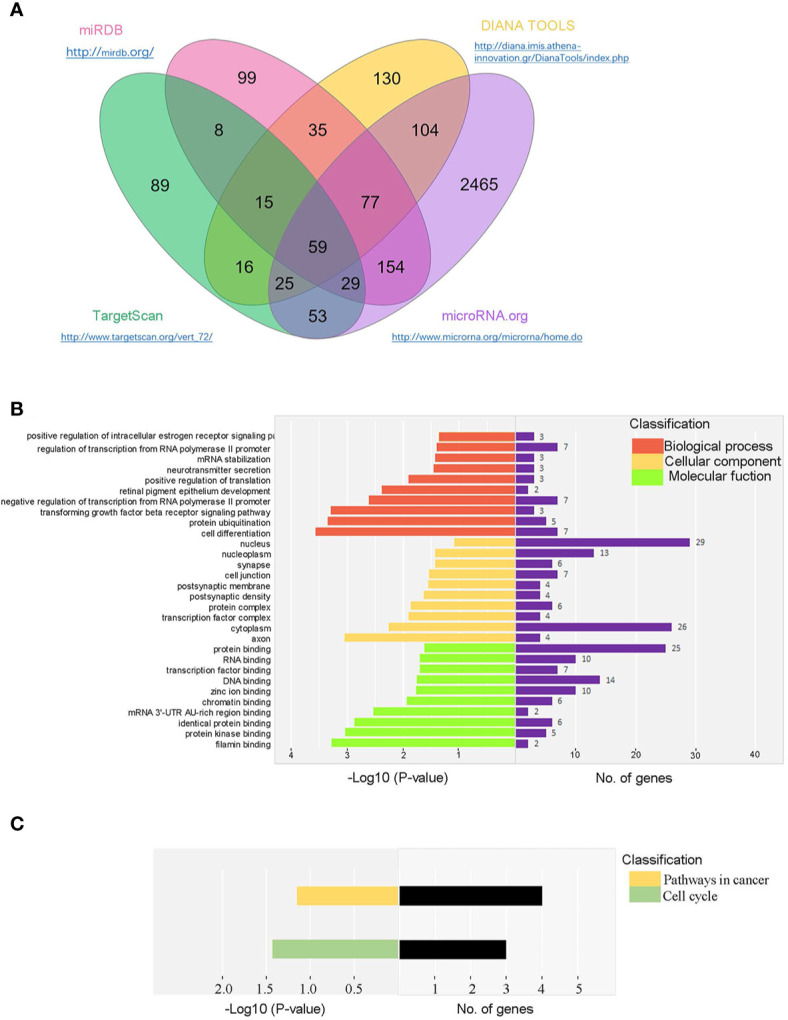
Bioinformatic prediction of miR-146a-5p target genes in mRTECs. **(A)** Venn diagram of identified miR-146a-5p target genes using multiple software programs (TargetScan, miRanda, miRDB, and DIANA-microT). Gene ontology (GO) analysis **(B)** and Kyoto Encyclopedia of Genes and Genomes (KEGG) analysis **(C)** of candidate genes in terms of biological process, cellular component, and molecular function.

We predicted the potential target genes of miR-146a-5p using multiple software programs (TargetScan, miRanda, miRDB, and DIANA-microT). Totally, 59 candidate genes were identified (**Figure 4A**). Next, gene ontology (GO) and Kyoto Encyclopedia of Genes and Genomes (KEGG) analysis were performed to evaluate mRNA enrichments in terms of biological process, cellular component, and molecular function. The top 10 enriched GO terms were involved in the regulation of cellular and metabolic processes (**Figure 4B**). KEGG analysis revealed two pathways, including the cell cycle and the pathways in cancer (**Figure 4C**), which are associated with cell activation and cell proliferation. Our results suggested that the differentially expressed miRNAs might be involved in the progression of renal fibrosis.

**Figure 5:**
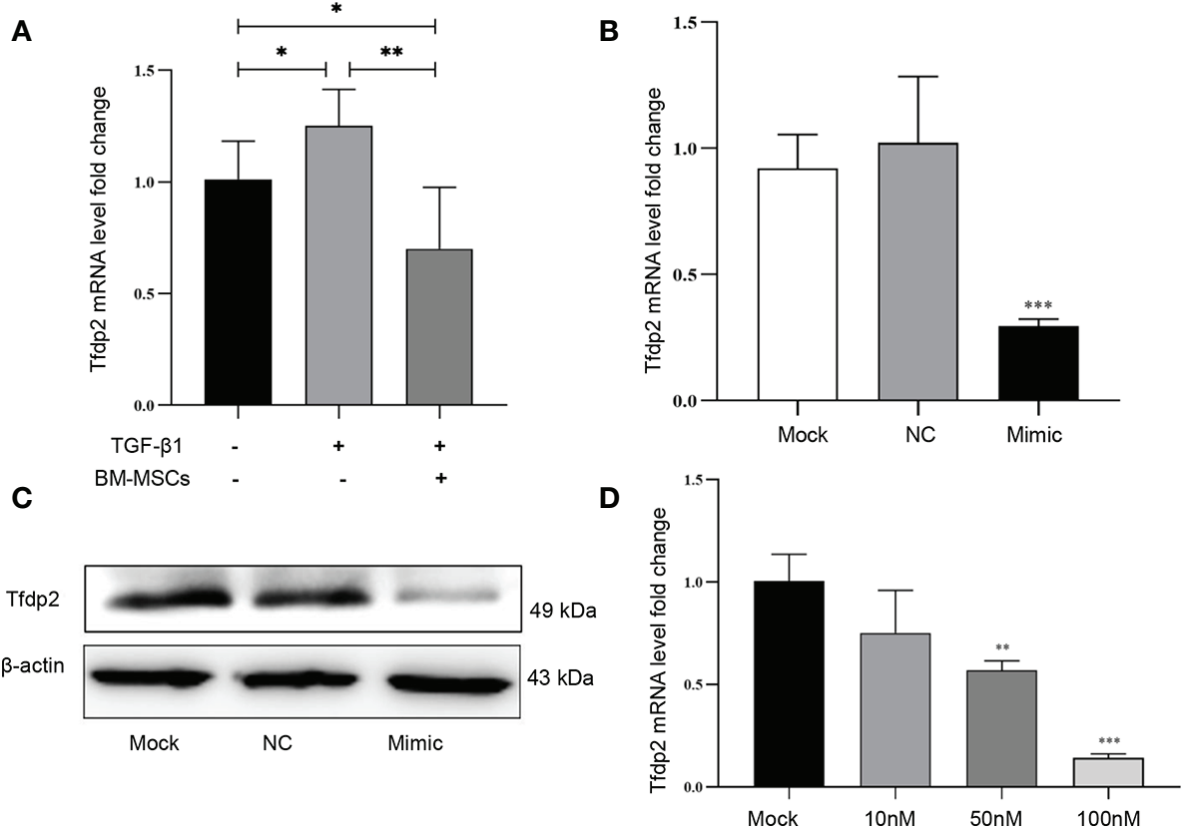
Verification of one candidate gene Tfdp2 in mouse renal tubular epithelial cells (mRTECs). **(A)** The levels of Tfdp2 were determined by real-time PCR in TGF-β1-treated mRTECs with or without bone marrow mesenchymal stem cells (BM-MSCs). The relative mRNA expression levels **(B)** and the protein levels **(C)** of Tfdp2 in mRTECs transfected by miR-146a-5p mimics were evaluated by real-time PCR and Western blot, respectively. **(D)** The transcript levels of Tfdp2 in mRTECs transfected by miR-146a-5p mimics with different concentrations. Data represent the mean ± SE from three independent experiments. *P < 0.05; **P < 0.01; ***P < 0.001.

Next, six candidate target genes of miR-146a-5p were selected from the KEGG pathway analysis. Expression of one candidate gene Tfdp2 was increased by exogenous cytokine TGF-β1 stimulation and decreased after co-cultured with BM-MSCs in mRTECs (**Figure 5A**). Whereas the expression of the other five candidate target genes (Smad4, Traf6, Rbl1, Ar, Rarb) revealed no statistical significance (**Supplementary Figures 1A–E**). The experimental data confirmed Tfdp2 gene as a candidate target gene of miR-146a-5p. To explore the direct relationship between Tfdp2 and miR-146a-5p, the expression of Tfdp2 mRNA and protein in mRTECs transfected by miR-146a-5p mimics was detected by RT-qPCR and western blot analysis. Our data showed that both mRNA and protein levels of Tfdp2 were decreased significantly compared with control (**Figures 5B, C**). Moreover, the mRNA level of Tfdp2 was gradually decreased depending on the miR-146a-5p mimic transfection concentration (**Figure 5D**). In summary, our experimental data indicate that Tfdp2 is the target gene of miR-146a-5p.

### Expression and Function of Tfdp2 in Renal Fibrosis

**Figure 6:**
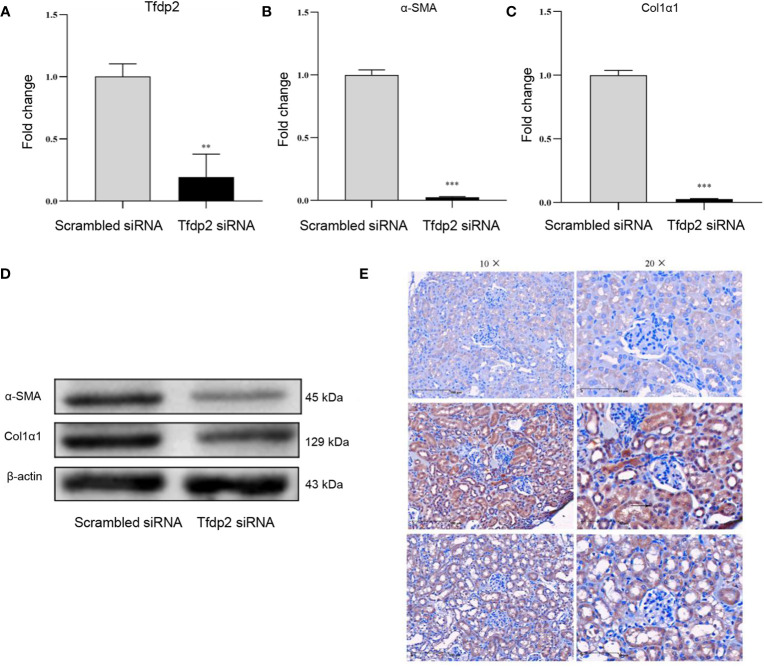
Overexpression of Tfdp2 in cisplatin-induced renal fibrosis. **(A)** The transcript levels of Tfdp2 in mouse renal tubular epithelial cells (mRTECs) was down-regulated by siRNA. The relative mRNA expression levels **(B, C)** and the protein levels **(D)** of α-SMA and Col1α1 after knockdown of Tfdp2 were by real-time PCR and Western blot, respectively. **(E)** Representative images of Tfdp2 immunohistochemical staining in cisplatin (CP)-induced mice kidney with or without bone marrow mesenchymal stem cells (BM-MSCs) treatment. Data represent the mean ± SE from three independent experiments. **P < 0.01; ***P < 0.001.

To evaluate the expression and function of Tfdp2 in renal fibrosis, we first transiently transfected Tfdp2 siRNA into mRTECs to down-regulate the expression of Tfdp2 in mRTECs (**Figure 6A**). Both transcriptional and translational levels of α-SMA and Col1α1 in mRTECs were significantly reduced by the knockdown of Tfdp2 (**Figures 6B–D**). These results suggested that inhibition of Tfdp2 expression can reduce the expression of fibrosis-related proteins. IHC analysis showed that a weak staining of Tfdp2 was predominantly localized to the cytoplasm in the normal tubulointerstitial compartment which was utilized as a reference. Stronger staining of Tfdp2 in renal tubular epithelial cells was observed in the renal tissues of the cisplatin group as compared with the control group, whereas the expression of Tfdp2 was obviously reduced in cisplatin + BM-MSCs group as compared to the cisplatin group. Taken together, these experimental data indicated that Tfdp2 exerts a significant function in the treatment of BM-MSCs on cisplatin -induced renal injury and fibrosis.

## Discussion

MSCs-based therapeutic approaches have been successfully applied to attenuate organ injury, including the kidney. Kidney fibrosis is a common endpoint outcome of CKD, which has a poor clinical outcome (19, 20). BM-MSCs are extracted from bone marrow and are defined as multipotent cells due to the robust capacities of differentiating into distinct functional cells. Most data reveal that BM-MSCs repair renal injury processes by the means of indirect secreted biomaterial, including the extracellular vesicles and multiple miRNA particles (21). In the present study, we established a model of cisplatin-induced renal fibrosis in C57BL/6 mice and found that BM-MSCs have an anti-renal fibrosis effect and renoprotection *in vivo*. Moreover, we showed that BM-MSCs significantly reduced the levels of α-SMA and Col1α1 in mRTECs treated by TGF-β1 in an indirect co-culture environment. Mechanically, we have identified miR-146a-5p as the most significant up-regulated miRNA in mRTECs regulated by BM-MSCs and plays a significant role in renal fibrosis in a co-culture experimental model. The results also found the gene Tfdp2 is one target gene of miR-146a-5p and exerts a vital role in the treatment of BM-MSCs on cisplatin-induced renal injury and fibrosis. Our study indicates that BM-MSC attenuates cisplatin-induced renal fibrosis by regulating miR-146a-5p/Tfdp2 axis in mRTECs.

Renal tubulointerstitial fibrosis is generally characterized by an excessive accumulation of extracellular matrix (ECM) factors (predominantly collagen type I) that prevent the regeneration of kidney tissue (22, 23). The severity of fibrosis notably correlates with the degree of kidney dysfunction and the renal failure outcome (24). TGF-β is well accepted as a critical regulator in renal fibrosis. Growing evidence indicates that exogenous cytokine, such as TGF-β1, can induce renal tubular epithelial cells to undergo phenotypic transformation into matrix-producing myofibroblasts (25, 26). Our study successfully established a mouse model to investigate the multiple low-dose cisplatin-induced renal fibrosis, which could be determined by increased levels of α-SMA and Col1α1 in mRTECs. In line with previous studies (11), we found BM-MSCs alleviate the severity of kidney dysfunction and fibrosis. Asanuma et al. (27). found arterially delivered MSCs protect against obstruction induced epithelial-mesenchymal transition (EMT) and chronic renal fibrosis involving alterations in TNF-α production. MSCs-based therapy was also reported to attenuate renal fibrosis through immune response modulation (28). However, the specific mechanisms of MSCs renoprotection remain poorly elucidative.

As a post-transcriptional regulator, miRNAs play an important role in the development, physiology, and maintenance of kidney microstructure. Dysregulation of miRNAs disrupts early kidney development, differentiation of kidney progenitor cells, and mature nephrons’ maintenance (29). Recent studies indicate that dysregulation of miRNAs is associated with the initiation and development of renal fibrosis in patients with diabetic nephropathy (DN) as well as in mouse models of DN (30, 31). Based on the function of BM-MSCs and miRNAs in renal fibrosis, we performed a high-throughput sequencing to identify the most significant regulated miRNAs by BM-MSCs in a co-culture experimental model. MiR-146a-5p was found as the most significant up-regulated miRNA, which was also verified by RT-PCR. Subsequently, we explored the function of miR-146a-5p in renal fibrosis *in vitro*. The gain- and loss-function of miR-146a-5p studies by mimic and inhibitor showed that the expression of α-SMA, Col1α1 mRNA, and protein were significantly altered in mRTECs. Therefore, our results indicate that BM-MSCs could inhibit the progression of renal fibrosis by regulating the expression of miR-146a-5p in mRTECs. Interestingly, a previous study has reported that miR-146a-5p acts as a negative regulator of TGF-β signaling in skeletal muscle after acute contusion, which indicates that miR-146 might have a therapeutic potential to alleviate skeletal muscle fibrosis (32). In addition, miR-146a-5p was found to inhibit the activation and proliferation of hepatic stellate cells (HSCs) in the progress of non-alcoholic fibrosing steatohepatitis (33). Therefore, miR-146a-5p exerts a critical function in the renoprotection of BM-MSCs.

Six candidate genes from the KEGG pathway analyses were confirmed by RT-qPCR. We found the transcription factor DP2 (Tfdp2) gene was increased in mRTECs stimulated by exogenous cytokine TGF-β1 and reduced after co-cultured with BM-MSCs. Consistent with miR-146a-5p targeting mRNA of Tfdp2, expression of Tfdp2 in mRTECs was significantly altered by the mimic and inhibitor of miR-146a-5p. To further investigate the function of Tfdp2 in renal fibrosis, Tfdp2 expression was knockdown by siRNA transfection. Both transcriptional and translational levels of α-SMA and Col1α1 in mRTECs were significantly reduced by the knockdown of Tfdp2. In addition, the relevance of Tfdp2 in renal fibrosis was confirmed in cisplatin-treated mouse kidney tissues by the IHC staining. Tfdp2 is a significant cofactor for the cell cycle control and gene expression, which combines with E2F family members to drive the function of c-Myc gene (34). The whole-genome analysis report for chronic kidney disease shows that Tfdp2 is related to renal function and CKD (35, 36). In the present study, we provided the first experimental evidence that BM-MSCs attenuate cisplatin-induced renal injury and fibrosis by the regulation of miR-146a-5p and the targeting gene Tfdp2, indicating that BM-MSCs may be a promising approach for developing novel therapeutics to treat renal fibrosis.

Nevertheless, our findings are only confirmed in the mouse model. The value and significance of BM-MSCs for the clinical treatment of renal fibrosis require more preclinical data support. In addition, the contribution of miR-146a-5p/Tfdp2 axis in renal fibrosis is worth further investigating and verifying in human kidney tissues.

## Conclusion

In conclusion, our findings demonstrate that BM-MSCs ameliorate renal injury and fibrosis *in vivo* and *in vitro*. Our study provides the first evidence that the protection of BM-MSCs on renal fibrosis *via* miR-146a-5p/Tfdp2 axis in renal tubular epithelial cells. These results offer novel mechanistic insights into the renoprotection of BM-MSCs, which highlights the promising clinical studies on MSCs-based therapy for kidney diseases in the near future. Additionally, the function of miR-146a-5p/Tfdp2 axis in renal fibrosis should be further investigated in preclinical and clinical studies, which might be a potential therapeutic target for renal fibrosis.

## Data Availability Statement

The datasets presented in this study can be found in online repositories. The names of the repository/repositories and accession number(s) can be found below: https://www.ncbi.nlm.nih.gov/geo/, GSE148144.

## Ethics Statement

The animal study was reviewed and approved by Experimental Animal Committee of Soochow University (Approval No: ECSU-201800099).

## Author Contributions

S-LW and M-MS conceived and guided the study. LW, QZ, XZ, and X-MZ performed the experiments. LW, CR, and SW analyzed and interpreted the data. LW and CR contributed to the writing and data presentation of the manuscript. All authors contributed to the article and approved the submitted version.

## Funding

This study was supported by the grants from the National Key R&D Program of China (No. 2018YFA0107500), Natural Science Foundation of Jiangsu Province (No. BK20200878), and the Priority Academic Program Development of Jiangsu Higher Education Institutions (PAPD).

## Conflict of Interest

The authors declare that the research was conducted in the absence of any commercial or financial relationships that could be construed as a potential conflict of interest.
