# Supplementary material for: Bone Marrow Mesenchymal Stem Cells Ameliorate Cisplatin-Induced Renal Fibrosis via miR-146a-5p/Tfdp2 Axis in Renal Tubular Epithelial Cells
Source: Front Immunol. 2021 Feb 16;11:623693. doi: 10.3389/fimmu.2020.623693 (PMC7921314; doi:10.3389/fimmu.2020.623693)
Supplement: Supplementary file 1 [file DataSheet_1.docx]

Supplementary Figure 1


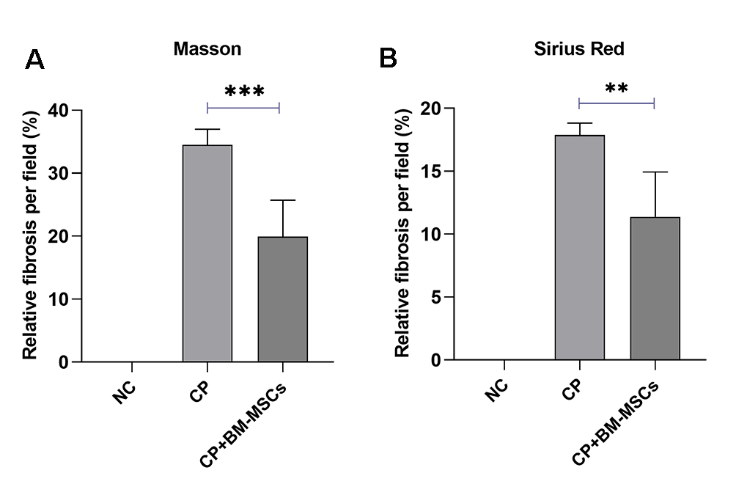


Supplementary Figure 1. Quantitative analyses of Masson (A) and Sirius red (B) staining in different groups. ***P*<0.01; ****P*<0.001.

Supplementary Figure 2


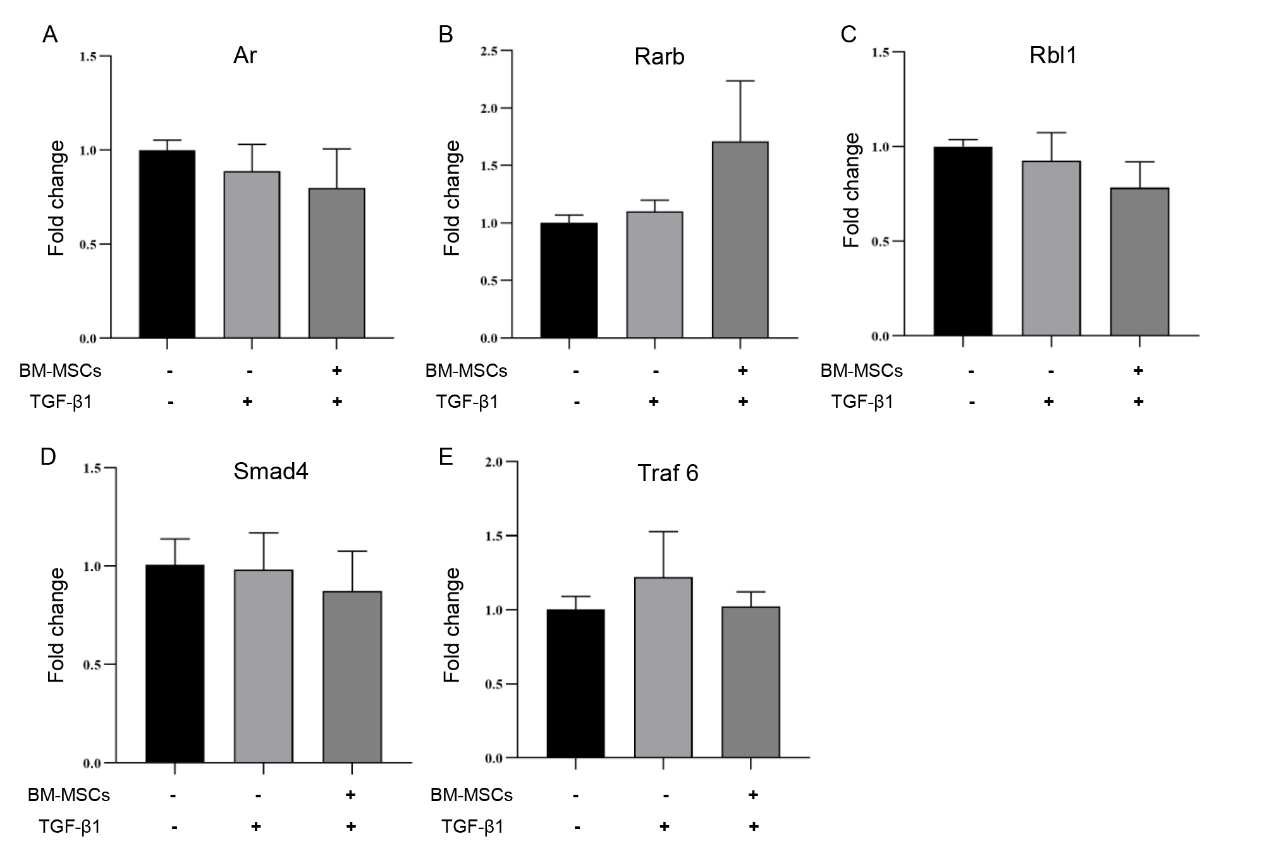


Supplementary Figure 2. The mRNA levels of candidate genes were determined by real-time PCR in TGF-β1-treated mRTECs with or without BM-MSCs.
